# Role of M1 macrophages in diabetic foot ulcers and related immune regulatory mechanisms

**DOI:** 10.3389/fphar.2022.1098041

**Published:** 2023-01-09

**Authors:** Yao Li, Xiaoyan Li, Shuai Ju, Wenqiang Li, Siyuan Zhou, Guili Wang, Yunmin Cai, Zhihui Dong

**Affiliations:** ^1^ Jinshan Hospital, Fudan University, Shanghai, China; ^2^ Zhongshan Diabetic foot Multidisciplinary Diagnosis and Treatment Center and Jinshan Operation Center, Shanghai, China; ^3^ Shanghai Medical College, Fudan University, Shanghai, China; ^4^ Zhongshan Hospital, Fudan University, Shanghai, China

**Keywords:** diabetes foot ulcer, immune infiltration, M1 macrophages, sequencing of single cell, gene associated with immunity

## Abstract

**Objectives:** Diabetes foot ulcers (DFUs) are characterized by immune infiltration of M1 macrophages observed in foot skin, in which immune-associated genes (IRGs) play a prominent role. The precise expression of IRGs as well as any possible regulatory mechanisms that could be present in DFUs is yet unknown.

**Methods:** The sequencing data of single-cell RNA (scRNA) in the foot skin of patients with DFUs were analyzed, screening out the cluster marker genes of foot skin obtained from the ImmPort database. IRG activity was assessed with the AUCell software package. The IRGs of DFUs were explored by analyzing the batch sequencing dataset of DFU skin tissue. HumanTFDB was adopted to identify relevant regulatory transcription factors (TFs). The STRING dataset was used to build the main TF protein–protein interaction networks. WB and immunofluorescence methods were used to verify M1 macrophage-related immune regulators.

**Results:** There were 16 clusters found: SMC1, fibro, t-lympho, he fibro, vasendo, baselkera, diffkera, SMC2, M1 macro, M2 macro, sweet/seba, B-Lympho, Melanio, lymphendo, plasma, and Schwann. M1 and M2 macrophages both had considerably higher AUC ratings than patients with DFUs compared to other sub-populations of cells. The proportion of M1 macrophages was the highest in the non-healing group. According to scRNA analysis and batch sequencing data by GO and KEGG, DEGs were enriched in immune response. Some 106 M1 macro-IRGs were finally identified and 25 transcription factors were revealed as associated with IRG expression. The PPI network indicated NFE2L2, REL, ETV6, MAF, and NF1B as central transcription factors.

**Conclusion:** Based on the bio-informatics analysis of scRNA and high-throughput sequencing data, we concluded that M1 macrophages may serve as the influencing factor of DFUs’ non-union. In addition, NFE2L2 could be involved in the regulation of IRG expression within M1 macrophages.

## Introduction

Diabetic foot ulcers (DFUs) are treated as a serious complication of diabetes mellitus (DM) (Miranda et al., 2021). They attack about 15%–25% of DM patients, resulting in a rate of lower limb amputation of L0-15 times higher than that of non-DM patients (Adem et al., 2020). DFUs are clinically characterized by numbness and pain of the limbs, destruction of bone structures, joint deformities, ulcers, and gangrene (Lauri et al., 2020). Currently, vascular disease and neuropathy are considered the primary causes of infection, while the specific etiology remains unclear (Theocharidis et al., 2020). Thus far, the main treatment methods for DFUs focus on night dressing, negative pressure, electrical stimulation, hyperbaric oxygen, and skin transplantation, although their outcomes are generally unsatisfactory (Chen et al., 2021). The annually increase in amputations also motivates an improvement in DFU treatments. Due to the ulcers being primarily disrupted by multiple risk factors, DFUs are pathologically complex and may be affected by poor patient adherence to treatment, the severity of ulcers, the location and duration of ulcers, vascular status, control of glycosylated hemoglobin (HbA1c) levels, smoking habits, and renal dysfunction (Dumville et al., 2017). These factors have led to an urgent requirement for novel clinical effective interventions to tackle this life-threatening disease. In DM patients, due to disorders of sugar, fat, and protein metabolisms, arterial wall atherosclerosis occurs 10–15 years earlier than that of healthy people (Wu et al., 2022). Plaque formation can be seen in the vascular wall of the lesion, and there are lipid deposits such as cholesterol in the plaque which narrow and block blood vessels. Because oxygen-carrying blood circulates in the arteries, a narrowing of blood vessels to organ tissue limits oxygen supply. Clinical symptoms only occur when blood vessels are narrowed by 75% (Brocco et al., 2018). However, it takes about 10 or even 20 years for the stenosis to change from mild to severe. If the patient’s blood glucose is not controlled, vascular wall lesions will continue to silently develop (Spanos et al., 2017). During this long period, the patient will not feel anything, and it will be at an advanced stage when symptoms manifest.

Studies have demonstrated that the macrophage phenotype may be a potentially effective treatment target for DFU, as hyperglycemia could increase the ratio of proinflammatory M1 macrophages to pro-regenerative M2 macrophages (Aitcheson et al., 2021). Manipulating the balance between M1 and M2 macrophages could be an effective approach for DFU treatment (Lin et al., 2022). IRGs, or immune-associated genes, are essential for immunological infiltration. Nevertheless, it is still unknown how IRGs manifest themselves in DFU and what possible mechanisms could control immune infiltration (Rong et al., 2022). Therefore, we used single-cell RNA (scRNA) as well as bulk sequencing data in a bio-informatics study to investigate the transcription properties and potential regulation mechanisms of IRGs within DFUs.

## Approaches

### Analysis of information from scRNA sequencing

Single-cell transcription data were obtained from the GEO database; construction of the GSE165816 dataset was based on the GPL24676 platform. The Seurat package (version 4.0.2) in R software was taken for cell cluster, following t-distribution randomized neighbor embedding (t-SNE) analyses plus principal component analysis (PCA). After cells with <200, >2,500, or >5% mitochondrial genes were filtered out, 32,574 filter cells were chosen for study. Logarithmic normalization was performed to normalize the gene expression, which was further scaled. The “VST” method was then used on every specimen to identify 2000 hypervariable genes (HUGs). Following that, principal component analysis was carried out to identify important principal components (PC), with the JackStraw and ScoreJackStraw functions used to visualize *p*-value distribution. Batch correction was achieved using the R package “Harmony” (edition .1.0). Based on these results, 18 PCs were used for the t-SNE testing. The cells were grouped using the FindCluster function according to 0.2, 0.3, 0.4, and 0.5, respectively. Based on the results of every group, the final resolution was determined as 0.4, and the cells were classified into 21 distinguished clusters. The differentially expressed genes (DEGs) for every cluster were found using the FindAllMarkers function of Logfc.Threshold = 0.25 as a guide. The cell types were then manually annotated in accordance with other research.

### IRG score

Screening the DEGs of every cluster revealed IRGs from the ImmPort database (https://www.immport.org/shared/home); 1,509 IRGs inside the DEGs were then chosen for IRG rating using AUCell (Version 1.12.0). Based on a gene set enriching study, the AUCell R program ranked the pathways for every cell. To assess the percentage of the chosen 497 IRGs that are strongly expressed in every cell, gene expression rankings for every cell were created based on the area under the curve (AUC) scores of those IRGs. Cells with higher AUC values were those that expressed more genes from the gene set. The threshold for identifying cells with active gene sets was determined using the “AUCell explore Thresholds” tool. The ggplot2 R package (Version 3.3.5) was then used to map every cell’s AUC score to the UMAP embedding in order to display the active clusters.

### Processing data sequencing in bulk

GEOquery software was used to obtain the raw data for GSE80178 from the GEO database (Version 2.58.0). Limma software was used to compute DEGs (edition 3.46.0). Significantly dysregulated genes were defined as DEGs with an absolute logFC more than 1 and an adjusted *p*-value less than .05. With the help of the ggplot2 program, volcano and heatmap plots were created (edition 3.3.5).

### GO and KEGG analysis

Intersection for the M1 macrophage cluster in GSE165816 and DEGs in GSE80178 yielded 131 differentially expressed IRGs, which were then introduced into the online bio-informatics website (https://maayanlab.cloud/Enrichr/) and Kyoto Encyclopedia of Genes and Genomes (KEGG) for Gene Ontology (GO) analysis, and the first 10 pathways were screened out according to the P-score order .

### PPI network development

Using STRING (https://string-db.org), a network analysis of protein–protein interactions (PPIs) was conducted. The TSV format file was download, which was imported into Cytoscape software for visualization, and the Cytoscape program was employed to filter hub TFs using the cytoHubba plug-in.

### Analysis of the immune infiltration using CIBERSORT

By using the CIBERSORT algorithm, the expression data for GSE80178 were analyzed, and the proportion of 22 types of immune cells was determined. These immune cells included immature B cells, memory B cells, plasma cells, CD8+T cells, immature CD4+T cells, resting memory CD4+T cells, memory cells with activated function, follicular helper T cells, regulatory T cells, γ-incremental T cells, and resting NK cells. The R software’s vioplot tool was employed to compare the different immune infiltration levels of every immune cell between the two cohorts.

### Clinical specimen acquisition

The DFU cohort of 22 patients with DFUs admitted to our hospital between March 2021 and January 2022, comprising 12 men and 10 women with a mean age of 56.12 ± 12.93 years and an average diabetes duration of 3–24 years. All patients had no heart, liver, kidney, or malignant tumors, and none had ever had a cerebral hemorrhage, a cerebral infarction, or an acute myocardial infarction (AMI). The healthy control (HC) group consisted of 12 healthy people who received physical exams at our hospital within the same time frame. The average age of this group of nine men and three women was 56.12 ± 12.93 years. There was no discernible difference between the three groups in terms of age or gender ratio (all *p* more than .05). A heparin anticoagulant tube containing 10 ml of every subject’s fasted blood was collected from their cubital vein in the morning. The serum was then separated and kept at −80°C after being centrifuged for 20 min at 3,500 rotations per minute. Six cases of foot skin tissue from DFUs and six HC cases were chosen and fixed with formalin. Immunofluorescence detection was then used to identify the disease.

### Western blotting

After washing, the skin tissue blocks were cut into small pieces and placed in a homogenizing tube for thorough homogenization. The specimen tube was taken out after homogenization, and ice bath and shock were carried out to ensure complete cleavage of the tissue. After centrifugation, the supernatant was collected to form the total protein solution. We prepared separation glue, added TEMED, shook well, and poured the glue. After solidification, electrophoresis began, and the membrane was then transferred. The primary antibody was added and incubated overnight. The secondary antibody was added after washing. Following incubation and washing, chemical luminescence, development, and fixation were performed. The optical density score of the target band was examined using an alpha program processing system—the protein expression level.

### qRT-PCR

Using the TRIzol reagent, total RNAs from the plasma were extracted and reverse-transcribed into cDNA. The PCR reaction comprised of 40 cycles of denaturation at 95°C for 10 min, annealing at 60°C for 1 min, and extension at 95°C for 15 s. Glyceraldehyde 3-phosphate dehydrogenase (GAPDH) was utilized as the internal standard. The levels of related factor expression were determined using a PCR reaction with 40 cycles of denaturation at 94°C for 15 min, annealing at 55°C for 30 s, and extension at 70°C for 1 min. GAPDH was used as an internal reference. Each experiment was conducted a minimum of three times. Using the 2^−ΔΔCT^ (cycle threshold (CT)) formula, the relative concentrations of NFE2L2, REL, ETV6, MAF, and NF1B were calculated. All the primers needed were purchased from Servicebio.

### Immunofluorescence method

Using 13 antibodies coupled to either cyanine 3 (cy3) or cyanine 5 (cy5) and 4′,6-diamidino-2-phenylindole (DAPI), eight staining cycles were performed on FFPE tissue microarrays. The antibodies employed were NRF2, ETV6, RELB, and NF1B GB113808, GB112082, GB11985 and bs-11899R are the numbers of antibody primer information of NRF2, ETV6, RELB, and NF1B, respectively, provided by Servicebio Laboratories, Inc. provided fluorescent-labeled secondary antibodies for use in the research. The paraffin sections were removed, put in water, and repaired in a microwave-safe repair box using citric acid (PH6.0) antigen repair solution. The primary and secondary antibodies were applied to the sections after they had been gently dried, and a circle was drawn around the tissue with a tissue chemical pen. The sections were then incubated for 50 min at room temperature in the dark. The slides were submerged in PBS (PH 7.4) and subjected to three 5-min decolorization washings. After 5 min, the ring was treated with autofluorescence quencher and washed under running water for 20 min. Anti-fluorescence quenched tablets were then used to seal the DAPI counterstained nuclei. Afterward, the slices were examined using a fluorescence microscope, and pictures were recorded and gathered.

## Results

### ScRNA analysis of skin tissue in DFUs

**FIGURE 1:**
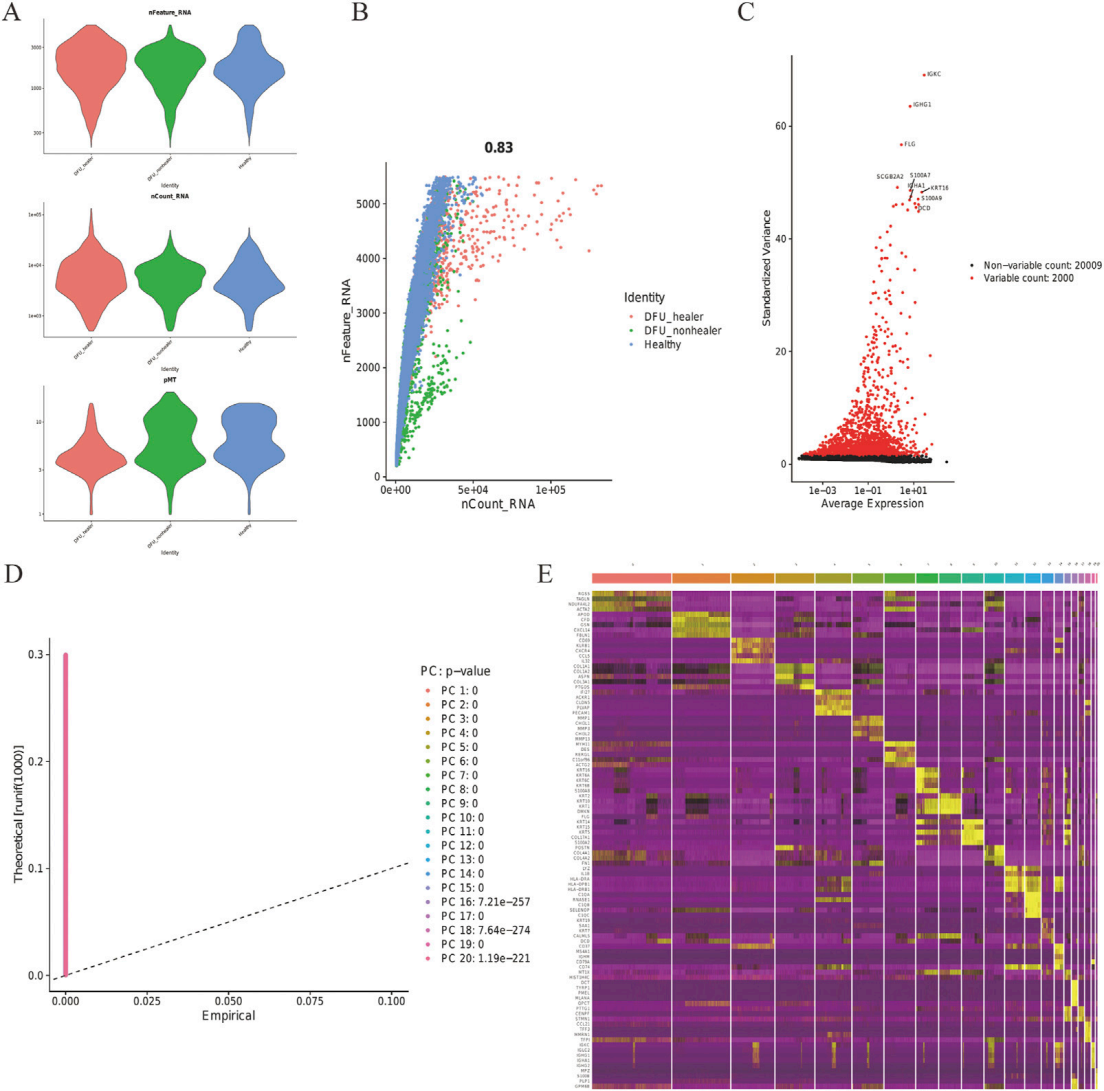
Skin tissue in DFUs was analyzed using scRNA. **(A)** Every specimen’s genes (features), numbers, and percentage of mitochondrial genes. **(B)** Correlation between the genes and counts for every specimen. **(C)** Top 10 HVGs were identified, and HVGs are colored in red. **(D)** JackStraw function selection of PCs. **(E)** Heatmap showing the top 10 DEGs within every cluster. Yellow labels are used to identify the top 10 DEGs.

The ScRNA sequencing dataset (GSE165816) was analyzed; after filtering, 22,009 feature analysis data of 32,574 cells were retained. Figure 1A shows the precise expression of every specimen. With a correlation coefficient of 0.83, the nCount RNA— representing the number of different molecular identifiers (UMI)—and the nCount_RNA— representing the number of genes—exhibited a positive association (Figure 1B). The top 10 HVGs were determined (Figure 1C). The top two HVGs—IGKC and IGHG1—are tiny calcium-binding proteins that are highly expressed in inflammatory situations. Using JackStrawPlot, all 20 PCs were detected by PCA with a *p*-value less than 0.05 (Figure 1D). Based on the previous research, the PCs were clustered with a selected number with the resolution determined, and all cells were finally divided into clusters. The differences of clusters before the annotation were analyzed, with the markers of all clusters determined. Eventually, 10 PCs were used to identify 16 clusters, and the first 10 DEGs for every cluster were presented (Figure 1E).

**FIGURE 2:**
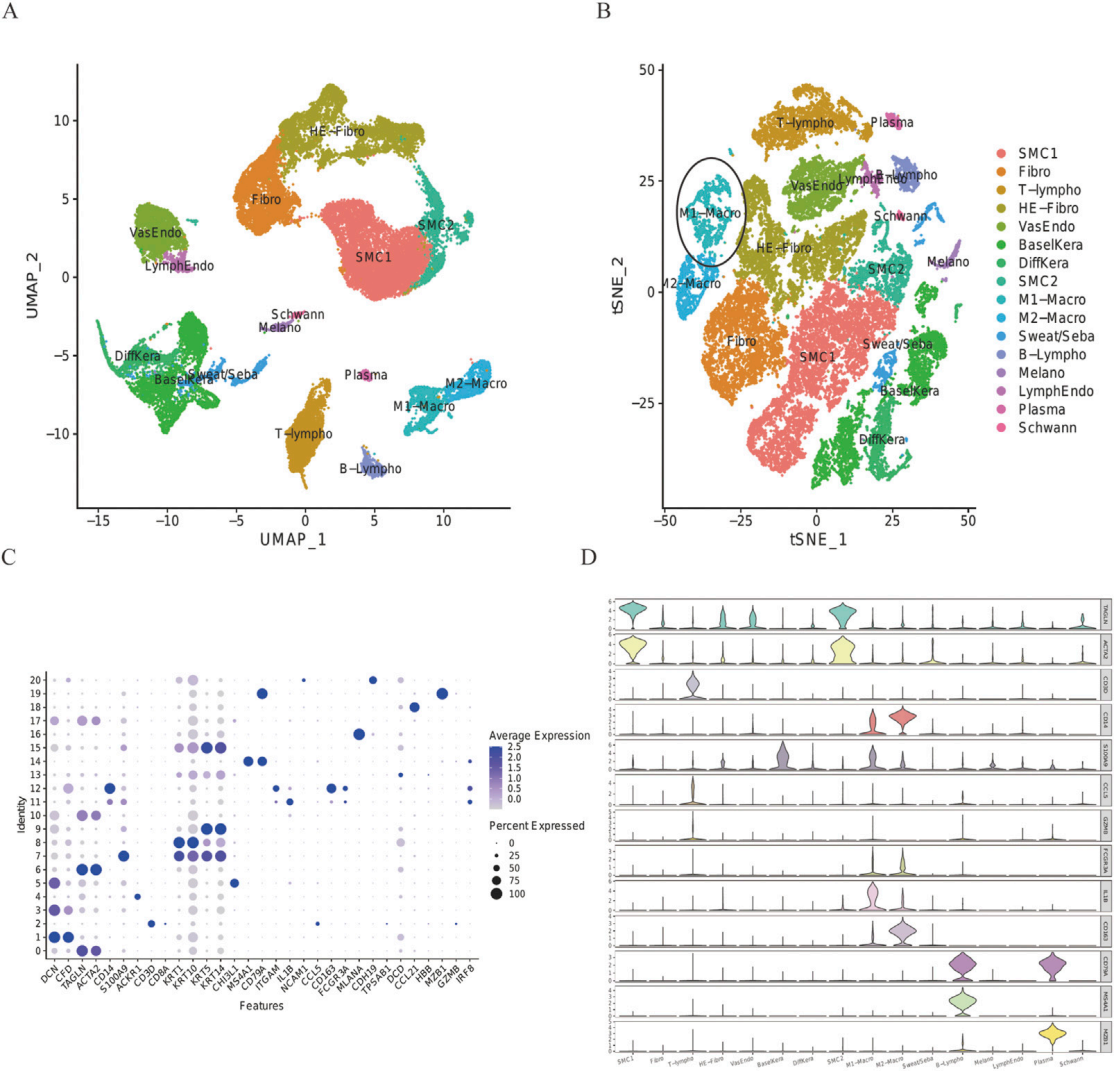
Expression of every cluster’s marker genes. **(A)** 32,574 cells from all scRNA were projected using tSNE. Different colors indicate different cell types. **(B)** DFU group’s tSNE projection. Ellipsoidal labeling was used to identify M1-Macro cells, and the number of these cells dropped in the DFU group. **(C)** Cell-type marker gene dot plot. The genes for cell-specific markers were chosen based on earlier research. The size and color of the dots indicate the average percentage of cells that express the chosen gene, respectively. **(D)** Violin plot uses density curves to show how the cell-type marker genes are distributed within every cluster. Every violin plot’s breadth reflects the proportion of cells with the appropriate level of gene expression.

T-SNE analysis allowed the 16 clusters to be visualized (Figure 2A). The proportion of M1-Macro clusters in the DFU group decreased in the ellipse-designated M1-Macro clusters (Figure 2B). The dot diagram (Figure 2C) plus violin chart shows the expression of cell-type marker genes (Figure 2D).

### IRG score of the skin cell group in patients with DFUs

**FIGURE 3:**
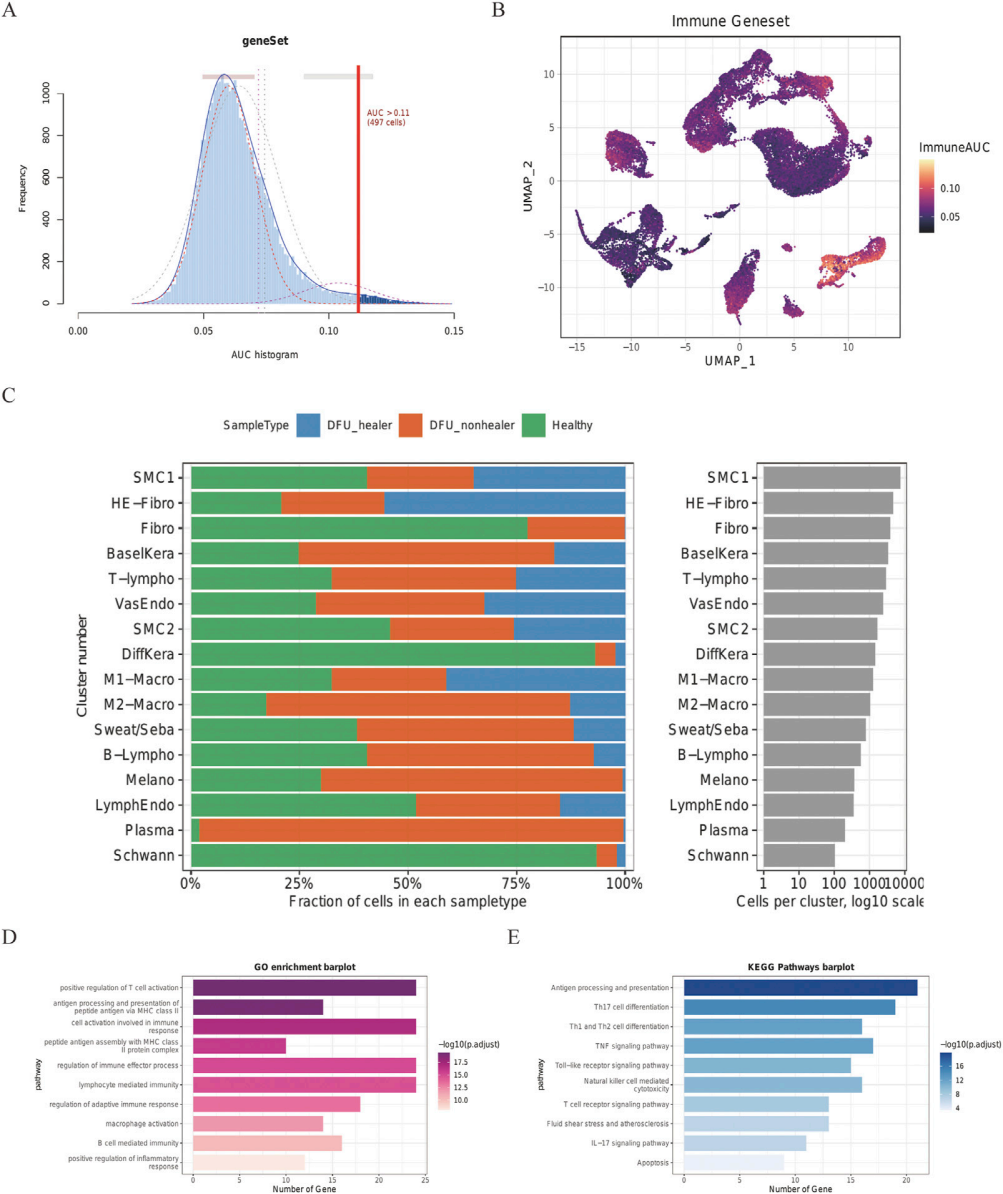
Skin cell group IRG score in DFU patients. Score from 212 screened IRGs. **(A)** Cutoff point was set at 11. **(B)** t-SNE chart showing the IRG results for every group. More genes were expressed by M1- and M2-Macro cells, which also had higher AUC values. **(C)** Quantity and makeup of every specimen’s cells. **(D)** DEGs in M1-Macro cluster GO assessment. **(E)** DEGs in M1-Macro cluster KEGG analysis.

The DEG of every cluster was screened according to the imported database to generate IRG and explore the IRG expression characteristics of skin cell groups in patients with DFUs. The IRGs in published studies were summarized in this database, and 212 IRGs were involved from the DEG of every cluster in the skin cell group. Using the AUCell R program, the IRG activity of every cell line was assessed (Figure 3A), and the AUC score of M1-Macro and M2-Macro was found to be significantly higher than other sub-populations of cells. Cells with more gene expression showed higher AUC values, which were mainly located in M1-Macro and M2-Macro in yellow in Figure 3B. Every specimen’s cell count and percentage are shown in detail in Figure 3C. Compared to the healing group, M1-Macro clusters were significantly reduced in the non-healing group (20% vs. 71%). In the subsequent analysis, we focus on M1-Macro clustering was mainly focused on (Supplementary Table S1). We also conducted GO and KEGG enrichment assessments on M1-Macro differentially expressed IRGs from skin tissue, which is mainly concentrated in immune-related pathways (Figures 3D,E).

### DFU DEGs with bulk sequencing information

**FIGURE 4:**
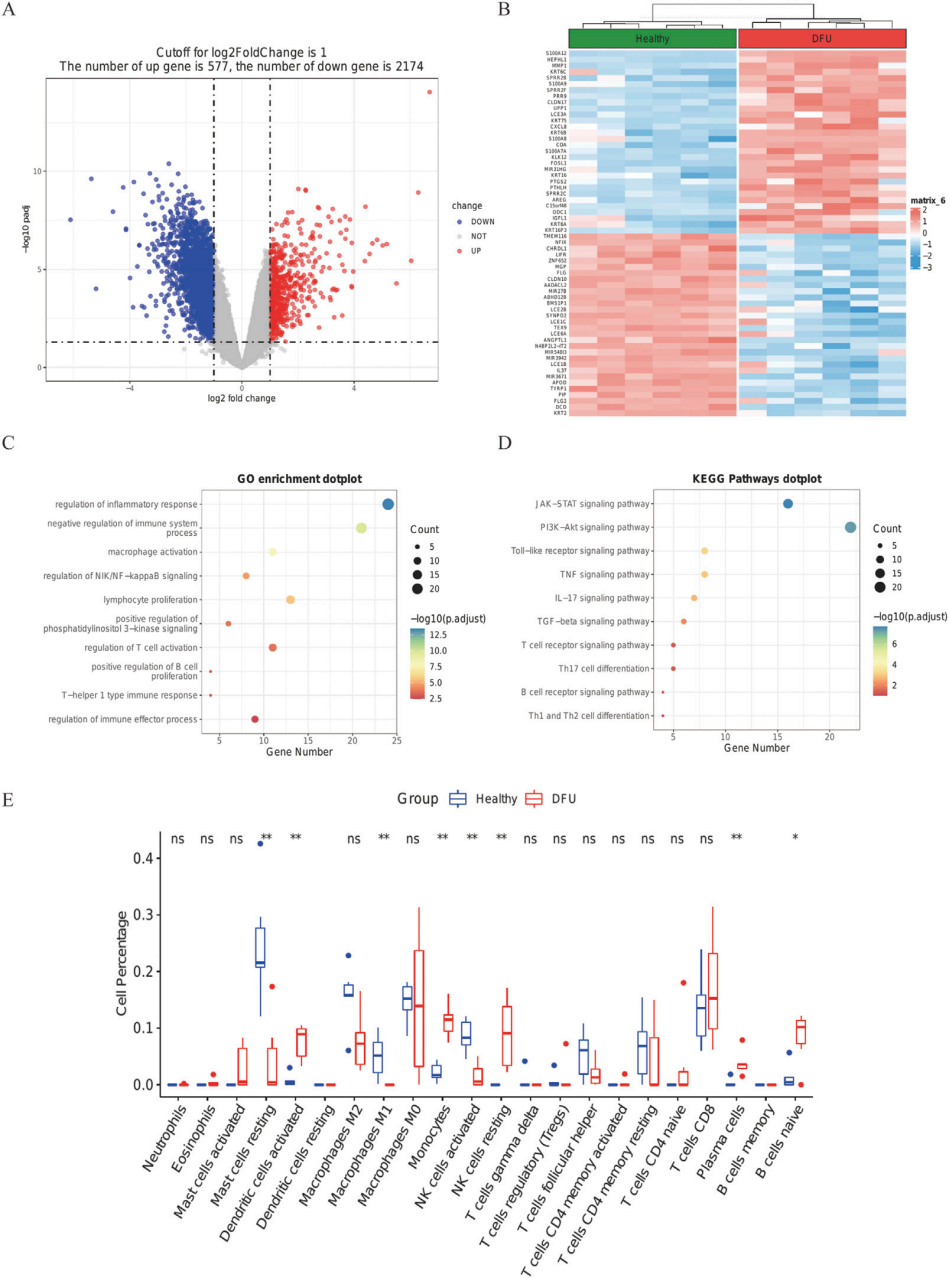
DFU DEGs from the GSE80178 database. **(A)** Volcano plot of DEGs (modified *p*-value .05; |logFC| >1). Red indicates up-regulated genes, and blue indicates down-regulated genes. **(B)** Heatmap showing GSE80178’s top 30 up-regulated DEGs and top 30 down-regulated DEGs. **(C)** GO of DEGs in GSE80178. **(D)** KEGG of DEGs in GSE80178. **(E)** Analysis of immune infiltration based on CIBERSORT (*p* < .05).

In terms of the expression characteristics of DFUs skin tissue, the high-volume RNA sequencing dataset GSE80178 from six foot-skin DFUs, three forearm-skin DFUs, and three control subjects were analyzed; the immune-associated biomarkers of DFUs were screened out by differential analysis. Those with | logfc | > 1 and corrected *p*-values less than 0.05 DEG (Supplementary Table S2) were chosen. A total of 577 up-regulated and 2,174 down-regulated DEGs in total were kept in DFUs (Figure 4A). The top 30 up-regulated DEGs and the top 30 down-regulated DEGs are shown as heat maps to display a relative consistency among groupings (Figure 4B). Interestingly, the top 10 GO keywords of DEGs in DFU skin large data were likewise centered on immune responses, which is congruent with the expression features of the M1-Macro cluster in DFU single cells (Figures 4C,D). The outcomes suggest a potential role for M1 macro-cells in foot skin tissue from DFU patients.

### Immune infiltration analyses

By running the CIBERSORT algorithm, the difference in immuno-osmosis between DFU and healthy skin tissues was assessed within 22 immune cell subtypes. Compared with healthy tissues, the number of M1 macrophages in DFU specimens were significantly low, which confirmed the conclusions of single-cell analysis (Figure 4E, *p* < 0. 05).

### Applicable regulatory transcription factors and common IRGs

**FIGURE 5:**
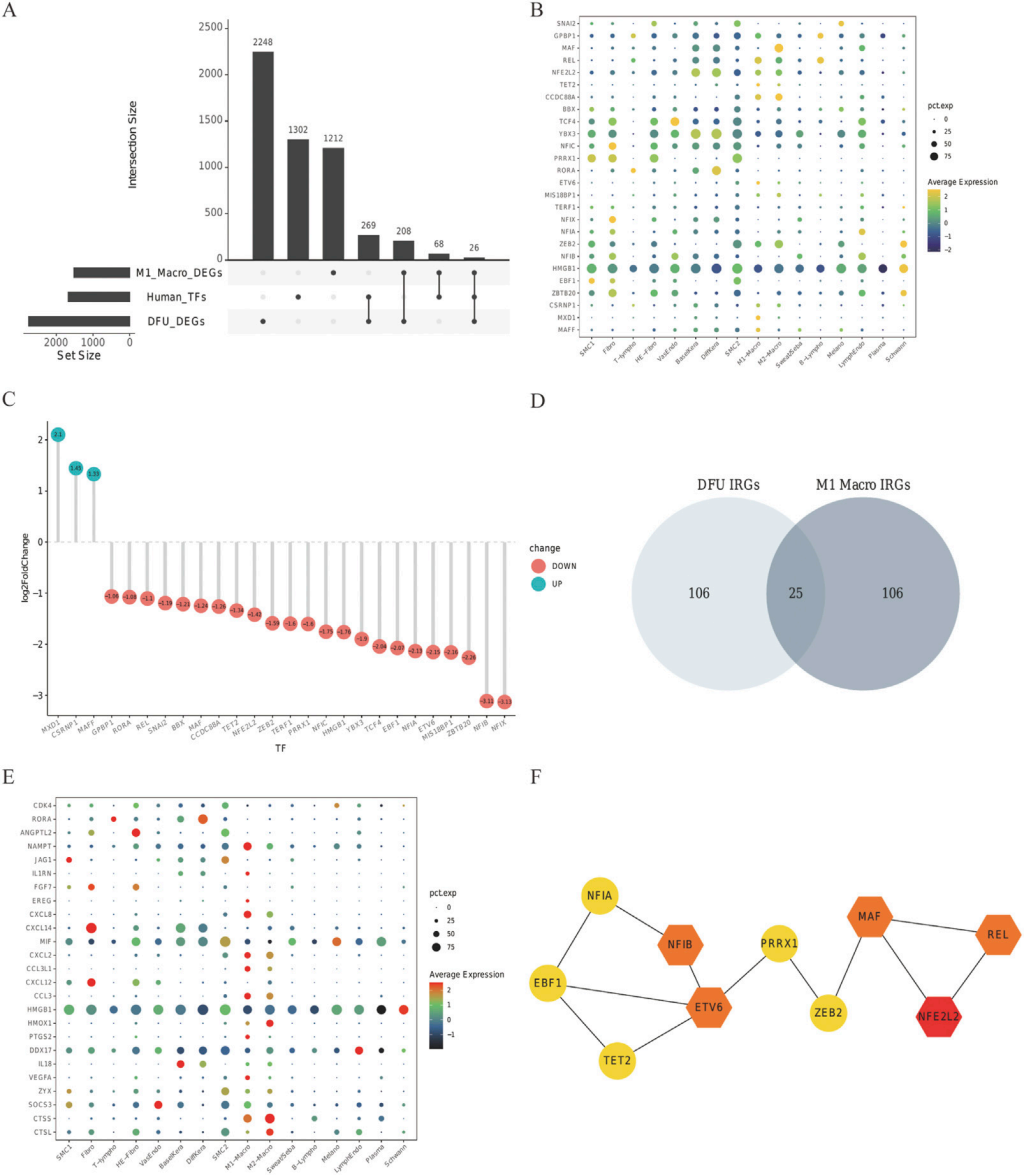
Relevant regulatory transcription factors and common IRGs. In the **(A)** upset plot, TFs in DFU in DEG of GES80178 and TFs in the M1 macrophage cluster from GSE165816, Human TF database, were shown. **(B)** GSE165816’s 26 TF expression levels. Common TF expression in the DEG of GES80178. **(C)** Green indicates up-regulated TFs, whereas red indicates down-regulated TFs. **(D)** DFU IRG and M1-Macro IRG Venn diagrams. The expression of shared IRGs between DFU and M1-Macro IRGs is shown in **(E)**. **(F)** STRING-illustrated PPI network of the common TFs. NFE2L2, REL, ETV6, MAF, and NF1B are used as hub genes.

We subsequently investigated the co-expression features of IRGs, taking into account the GO for IRGs in IRGs and DFU skin—which is mostly focused on immunological response in the skin tissue cell M1 macrophage cluster and DFU skin. Some 1,665 TFs were obtained from HumanTFDB to study the transcriptional regulatory activity of IRGS. A total of 26 TFs were acquired by intersecting with the MI macrophage cluster and IRGs in DFU skin (Figure 5A). The default arguments for the FindAllMarkers function were only.pos is true, min.pct is equal to 0.25, and logfc.Threshold is equal to 0.25. The expression of the 26 TFs in GSE165816 and GSE80178 is shown in Figures 5B,C, among which three factors were up-regulated in DFUs, and all 26 TFs were expressed in M1 macrophages. The different IRGs of the scRNA analysis portion of the M1macro and the bulk RNA-seq part were intersected to obtain 25 common IRGs (Figure 5D and Supplementary Table S3). Their expression is shown in Figure 5E. The 26 co-expressed TF were input into STRING database to construct a PPI network. The cytoHubba plug-in in Cytoscape was analyzed by hub, with the different methods ranked as follows: MultiClass Classification, DMNC, MN, Degree, EPC, EcIcentrity of Bottle Necks, Radiality of Closeness, Betweenness, the Stress factor, and ClusteringCoefficient. The top five with a relatively greater frequency were selected hubTFs for constructing the PPI network. NFE2L2, REL, ETV6, MAF, and NF1B may play a key role in the transcriptional regulation of IRGs as central genes (Figure 5F).

### Immune-associated genes are associated with a relative immune microenvironment for DFUs

**FIGURE 6:**
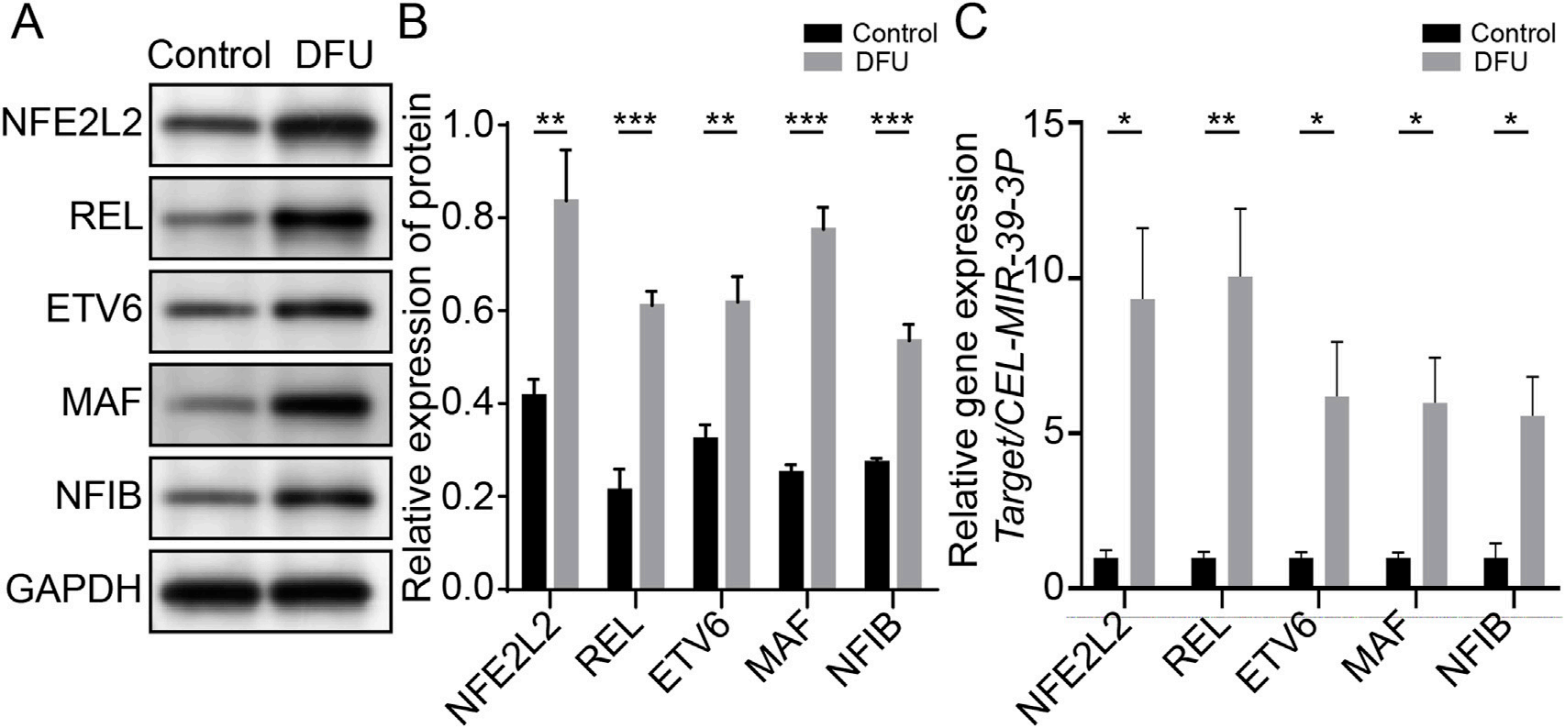
Immune-associated genes are associated with relative immune microenvironment for DFUs. **(A)** Native strips of NFE2L2, REL, ETV6, MAF, and NF1B in WB. **(B)** Blood of DFU patients showing significantly higher expression levels of NFE2L2, REL, ETV6, MAF, and NF1B. **(C)** qPCR results showed that NFE2L2, REL, ETV6, and NF1B in the DFU group were significantly higher than those in the control group.

Plasma tissues from 22 DFUs patients and 12 healthy subjects were clinically collected to further support the hypothesis that the genes screened previously are associated with the immune microenvironment of DFUs. The expression levels of NFE2L2, REL, ETV6, MAF, and NF1B genes were first discovered in the blood by WB and qPCR. We noticed that the blood of DFU patients had significantly higher expression of NFE2L2, REL, ETV6, MAF, and NF1B (Figures 6A,B). qPCR analysis of patients’ blood pressure also showed that immune-related factors were significantly higher than those of healthy people, with a statistical significance (Figure 6C and Supplementary Table S4).

**FIGURE 7:**
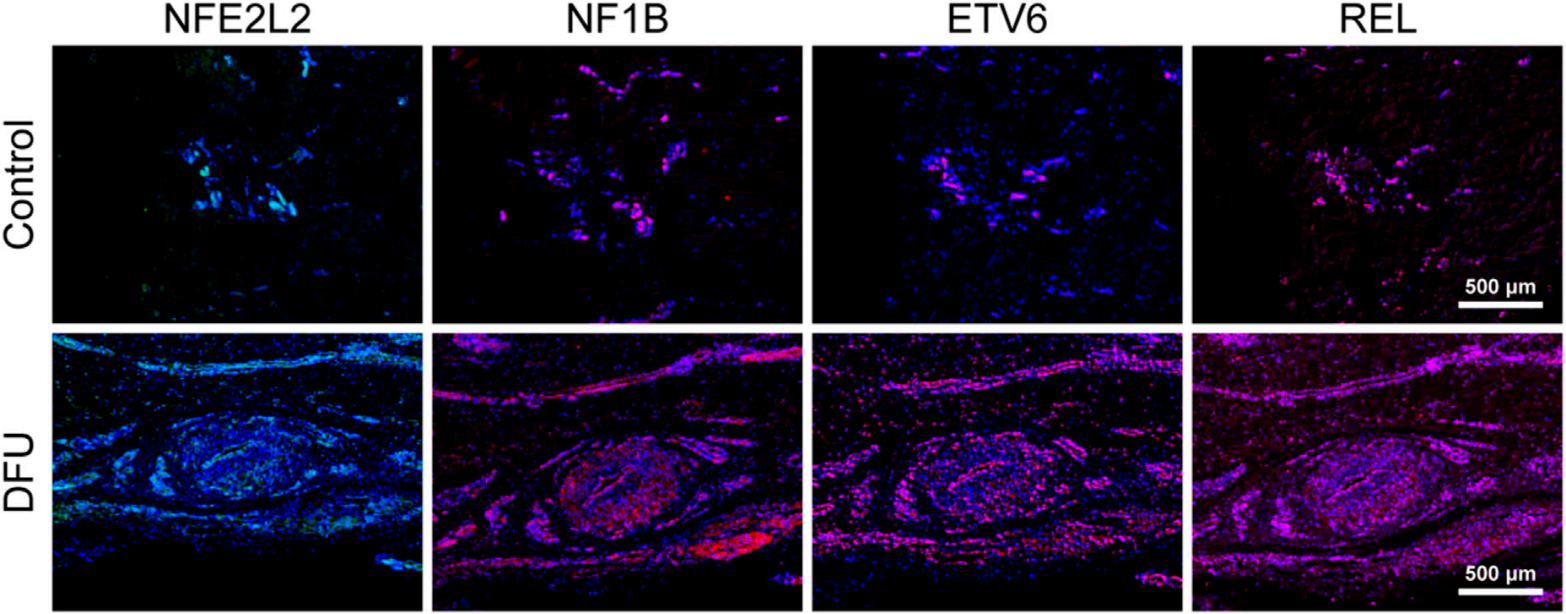
Immune-associated genes are associated with the relative immune microenvironment for DFUs.

A number of immune-associated genes were also identified in skin tissue taken from six DFU patients and healthy subjects using immunofluorescence. The expression of NFE2L2, REL, ETV6, and NF1B in skin tissues of healthy subjects and DFU patients were analyzed by labeling under a fluorescence microscope. The findings demonstrated that NFE2L2, REL, ETV6, and NF1B transcription in DFU skin was significantly higher than that in healthy human skin. This was consistent with the results of our single-cell sequencing, which showed that IRGs may be involved in the immune function of DFUs (Figure 7).

## Discussion

Macrophages are mainly derived from hematopoietic stem cells and belong to the monocyte group of immune cells (Wculek et al., 2022); myeloid and lymphoid stem cells may develop from hematopoietic stem cells. The former will differentiate into monocytes, red blood cells, and platelets and the latter into T, B, and NK cells (Bain and Schridde, 2018). The distinct roles and inflammatory cytokine levels of M1 and M2 macrophages allow for their classification (Bardi et al., 2018). M1 macrophages (classical activated macrophages), which are largely activated by LPS and IFNγ, can release IL2 in high concentrations and IL10 in low concentrations, which principally promote inflammation, sterilization, and phagocytosis (Song et al., 2022). IL4 inflammatory cytokines are primarily responsible for activating M2 macrophages (alternatively activated macrophages), which inhibit M1 macrophages by secreting anti-inflammatory cytokines such as IL10 and thus play a role in wound healing and tissue repair (Hu et al., 2022). M1 macrophages could protect tissues and organs from invasion of foreign substances by mainly phagocytosing foreign substances such as bacteria and endogenous substances such as apoptotic cell debris *in vivo* (Locati et al., 2020). In inflammatory diseases, M1-type macrophages will be enriched in the early inflammatory sites and activated by proinflammatory factors, such as LPS, TNFα, and IFNγ, and then accelerate the occurrence and development of inflammation by secreting inflammatory factors such as IL12 to defend against invasion by foreign substances (Chiang et al., 2017; Atri et al., 2018; Orecchioni et al., 2019). M2 cells are conducive to inhibiting inflammation, repairing tissue, and rebuilding tissue structure in the later stage of inflammation. The number and proportion of M1/M2 macrophages will vary continuously with the passage of time in the development of inflammation, finally completely eliminating the impacts of inflammation (Witherel et al., 2021). However, in some chronic inflammation, specific acute validation processes will result in a dysregulated ratio of M1/M2 cell groups, where excessive M1 cell group activation may cause significant tissue injury and result in a more severe inflammatory cytokine storm and other adverse symptoms (Jiang and Li, 2022). Tumor-associated macrophage (TAM) refers to macrophages recruited to the tumor microenvironment (Borriello et al., 2022). These were thought to inhibit tumors in early studies, while subsequent studies revealed that TAM could promote tumor deterioration by suppressing immune response, promoting angiogenesis, and stimulating tumor cell infiltration and metastasis (Chen et al., 2019). As an essential component in the tumor microenvironment, TAM plays a crucial role in the structure and functionality of the tumor microenvironment. In the current study, most TAM cells in tumor microenvironment exhibited the characteristics of M2 macrophages with the high expression of IL10, which functioned by inhibiting the immune response in the tumor microenvironment (Shan et al., 2020). Subsequent studies on the tumor microenvironment have also proved that an imbalanced M1–M2 ratio plays a critical role in tumor growth and immune escape, and subsequent metastatic drug resistance (Xu et al., 2022).

In this paper, the cell proportion of the three types of specimens was calculated through data mining, followed by the composition of the cell proportion of every cell subset. This revealed that, compared to the healing group and the healthy cohort, the fraction of M1 macrophages within the non-healing cohort was much lower. In contrast, the proportion of M2 macrophages in the non-combined group increased. We speculated that this was a result of M1 macrophages not being able to activate during the occurrence of DFUs, and microorganisms causing severe ulcers in the skin tissue. However, the more numerous M2 macrophages did not contribute to inhibiting inflammation, repairing tissue, and rebuilding tissue structure. Thus, DFUs seemed more to be a refractory disease, for which regulating the polarization balance of macrophages (M1–M2) has been considered a therapeutic strategy. The most significant feature of this paper is that the expression levels of NFE2L2, REL, ETV6, MAF, and NF1B genes were first detected in blood by WB and qPCR. We noted that the expression levels of NFE2L2, REL, ETV6, MAF, and NF1B in the blood of DFU patients were significantly increased. qPCR analysis of patients’ blood pressure also showed that immune-related factors were significantly higher than those of healthy people, with statistical significance. Immunohistochemical results also supported the appeal findings. Therefore, the conclusion that immune-related factors are closely related to the microenvironment of DFUs is also positive. Despite recent advances in our understanding, macrophage polarization needs further in-depth exploration. 1) Macrophage polarization is a dynamic process, where current knowledge suggests that it restricts the monitoring fixed at a certain time and in a specific environment. How should dynamic monitoring be considered in the future, so as to better observe its variation? 2) Further study would be worthwhile to assess how the related pathways of macrophage polarization can be manipulated in order to provide a more solid theoretical basis for clinical practice. 3) DFUs may exhibit an acute attack stage, a plateau stage, and a recovery stage, corresponding to different levels of M1 macrophages. 4) In China, diabetes mellitus is more likely to result in vascular disease, while neuropathy is more likely in other countries. There may be differences in the number of M1 macrophages due to different lesion statuses; this requires verification by further experiments and provides an objective for clinical staging and treatment.

## Data Availability

The original contributions presented in the study are included in the article/Supplementary Materials; further inquiries can be directed to the corresponding author.
